# Not only the main streams, but also the tributaries supply the landscape: antidepressants for the prevention of severe COVID-19

**DOI:** 10.1007/s00406-025-02140-7

**Published:** 2025-11-10

**Authors:** Udo Bonnet

**Affiliations:** 1 Department of Mental Health, Evangelisches Krankenhaus Castrop-Rauxel, Academic Teaching Hospital of the University of Duisburg, Essen, Castrop-Rauxel, Germany; 2 Department of Psychiatry and Psychotherapy, Faculty of Medicine, Landschaftsverband Rheinland-Hospital Essen, University of Duisburg-Essen, Essen, Germany

COVID-19: Since the majority of the population has now acquired a certain degree of immunity through infection and/or vaccination, severe acute cases are rare compared to the beginning of the pandemic. The likelihood of severe COVID-19 increases with age and comorbidity including severe mental illness [1]. SARS-COV2 is now an endemic virus also in Germany, with COVID-19 peaking in autumn and winter. The British Journal of Medicine has just published the most scientifically sophisticated metaanalysis to date on the treatment of COVID-19, which requires comment with regard to the role of antidepressants (AD) in the prophylaxis of severe COVID-19 [2]. Regarding AD, the most randomized studies on the treatment of COVID-19 have been conducted with fluvoxamine, which is inexpensive and easy available, even in developing countries [3]. The aforementioned recent meta-analysis found that fluvoxamine is not convincingly more effective than standard care for mild or moderate COVID-19 [2]. The antiviral drugs nirmatrelvir-ritonavir and remdesivir, both of which are approved for COVID-19 and therefore have high-quality clinical trials funded by the pharmaceutical industry, proved to be particularly beneficial in that metaanalysis [2]. Similiar trials are unlikely for substances like fluvoxamine that have been on the market for a long time and are approved for other non-viral diseases, as patent protection for the treatment of COVID-19 would be complicated to obtain and not very lucrative, resulting in less interest from the pharmaceutical industry to carry out randomized prospective two-arm (e.g. fluvoxamine versus approved medications for COVID-19) or even three-arm studies including a further placebo condition. Nevertheless, even cost-effective substances such as most ADs might have the ability to halt the development of severe COVID-19 (principle of repurposing drugs, e.g., for COVID-19) [4]. In this context, I think it is worth mentioning that fluvoxamine, but also other ADs, have the biological potential to limit the cytokine storm triggered by SARS-COV2 and thus theoretically prevent a severe course of COVID-19 if administered early enough in the course of the disease; incidentally, not only in early COVID-19, but also in other viral infections that lead to an “excessive immunological alarm situation” [3].

This hypothesis (concerning COVID-19) was tested in a series of large-scale retrospective studies (˃20000 participants/chart reviews), which could not be included in the network meta-analysis [2] for methodological reasons. Most of these large retrospective studies provided promising results for a couple of AD for the prevention of severe COVID 19 [3]. One of them even found a dose dependency [5]. In addition, there are some larger randomized trials with fluvoxamine, most compared to usual care or placebo plus usual care, with mixed results [2, 3]. To my knowledge, there is only one randomized study indirectly comparing fluvoxamine with an antiviral (fluvoxamine in combination with favipiravir versus flavipiravir alone), which showed no additional benefit of fluvoxamine [6], but no studies directly comparing fluvoxamine with drugs approved for mild to moderate COVID-19. Fortunately, the pandemic did not last long enough to realize such necessary studies to proof the real value of fluvoxamine or further ADs to prevent severe COVID-19. Therefore, the certainty of evidence (according to GRADE) for fluvoxamine to prevent severe COVID-19 will most likely remain only low, which is in line with the recent assessment [2].

In the run-up to the recent network metaanalysis, which now indicates the “*main stream*”, there were curiously more meta-analyses than randomized studies on fluvoxamine [3], which indicates the great interest in this topic. Most of these prior meta-analyses found add-on fluvoxamine to be beneficial in preventing severe COVID-19 with good tolerability and safety [3]. Among these, the most recent meta-analysis by Deng et al. also found a dose dependency [7].

Given the results of the large retrospective studies and several previous meta-analyses, which even provide evidence of dose dependency (“*tributaries*“), I believe that SARS-COV2-infected patients or those with early COVID-19 who are already taking fluvoxamine or other ADs for other conditions should not be denied the additional chance of a potential benefit against the development of severe COVID-19 by discontinuing the ADs. Rather, patients should be educated about the potential protective effect of ADs against severe COVID-19 [3, 8]. Severe COVID pneumonia does not appear to be positively or negatively affected by fluvoxamine [3]. A retrospective multicenter study found that adults who were already pre-medicated with AD for neuropsychiatric disorders at the time of COVID-19 onset had a significantly better chance of survival with ECMO therapy for COVID-19-related respiratory failure than affected patients without AD (N = 149, 22.0% vs. 6.5% survivors) [9].
